# Specific threonine-4 phosphorylation and function of RNA polymerase II CTD during M phase progression

**DOI:** 10.1038/srep27401

**Published:** 2016-06-06

**Authors:** Corinna Hintermair, Kirsten Voß, Ignasi Forné, Martin Heidemann, Andrew Flatley, Elisabeth Kremmer, Axel Imhof, Dirk Eick

**Affiliations:** 1 Department of Molecular Epigenetics, Helmholtz Center Munich, Center of Integrated Protein Science (CIPSM), Marchioninistrasse 25, 81377 Munich, Germany; 2 Biomedical Center Munich, Center of Integrated Protein Science (CIPSM), ZFP, Großhaderner Strasse 9, 82152 Planegg-Martinsried, Germany; 3 Institute of Molecular Immunology, Helmholtz Center Munich, Marchioninistrasse 25, 81377 Munich, Germany

## Abstract

Dynamic phosphorylation of Tyr1-Ser2-Pro3-Thr4-Ser5-Pro6-Ser7 heptad-repeats in the C-terminal domain (CTD) of the large subunit coordinates progression of RNA polymerase (Pol) II through the transcription cycle. Here, we describe an M phase-specific form of Pol II phosphorylated at Thr4, but not at Tyr1, Ser2, Ser5, and Ser7 residues. Thr4 phosphorylated Pol II binds to centrosomes and midbody and interacts with the Thr4-specific Polo-like kinase 1. Binding of Pol II to centrosomes does not require the CTD but may involve subunits of the non-canonical R2TP-Prefoldin-like complex, which bind to and co-localize with Pol II at centrosomes. CTD Thr4 mutants, but not Ser2 and Ser5 mutants, display severe mitosis and cytokinesis defects characterized by multipolar spindles and polyploid cells. We conclude that proper M phase progression of cells requires binding of Pol II to centrosomes to facilitate regulation of mitosis and cytokinesis in a CTD Thr4-P dependent manner.

Eukaryotes express three different DNA dependent RNA polymerases, Pol I, Pol II, and Pol III. All polymerases are highly related and have identical and similar subunits in common^1^. However, the large subunit (Rpb1) of Pol II has evolved a unique carboxy-terminal domain (CTD) consisting of an unstructured tail of heptad-repeats with the consensus sequence Tyr1-Ser2-Pro3-Thr4-Ser5-Pro6-Ser7. The length of this tail varies from 26 repeats in yeast to 52 repeats in mammals^2^. The dynamic posttranslational modification and structural plasticity enable the CTD to serve as a binding platform for a variety of factors to regulate the transcription of chromatin templates and maturation of nascent transcripts. Phosphorylation of Ser, Tyr, and Thr residues^3^,^4^,^5^,^6^,^7^,^8^,^9^,^10^, cis/trans isomerisation of proline residues^11^,^12^,^13^, as well as the methylation and acetylation of Lys and Arg residues in non-consensus repeats^14^,^15^,^16^,^17^,^18^ induce local changes in CTD structure, and thereby regulate the interaction with factors of the transcription machinery^19^,^20^,^21^,^22^,^23^. The various modifications of CTD have been linked to specific stages of Pol II in the transcription cycle and occur in CTD heptad repeats in a coordinated manner^24^,^25^,^26^ supporting the model of a code underlying the various CTD modifications^20^,^27^,^28^.

Posttranslational modifications of CTD have been studied mainly in interphase cells while little is known about CTD modification during M phase. Pol II-transcribed genes are globally repressed at the level of initiation with the start of M phase. Genes carrying a paused or elongating Pol II continue and finish transcription at the end of genes^29^. However, recent studies reported specific Pol II transcription also in M phase cells. Pol II at centromeric regions is phosphorylated at CTD Ser2 and Ser5 residues, transcribes centromeric α-satellite RNA^30^,^31^, and supports targeting of regulatory factors (e.g. Sgo1) to compacted mitotic chromatin to allow coordinated chromosome segregation in mammalian cells^32^. Thus, while a large fraction of Pol II is no longer associated with chromatin after onset of mitosis a small fraction is recruited to centromeric sequences and remains transcriptionally active. Mitosis-specific transcription of Pol II has also been observed for intergenic spacer sequences of ribosomal genes and of subtelomeric sequences in *S. pombe*. Silencing of this transcription is achieved by removal of Ser2-P and Ser5-P marks of CTD by phosphatase Cdc14 and is a prerequisite for mitotic exit^33^.

Here we describe a further specific function of Pol II in M phase cells. Pol II phosphorylated at CTD Thr4, but not Tyr1, Ser2, Ser5, and Ser7 residues, specifically associates with centrosomes and the midbody in M phase cells. This association is critical for coordinated M phase progression since expression of Thr4 mutants, but not of other CTD mutants display severe defects in M phase progression and proper segregation of chromosomes.

## Results

### M phase-specific, Thr4 phosphorylated, high molecular weight form of Pol II

**Figure 1:**
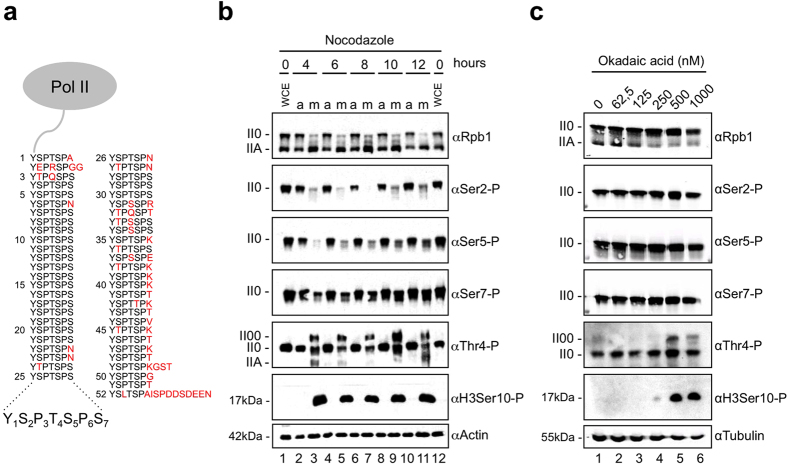
Thr4-P levels are subject to changes in mitotic cells. (**a**) Pol II large subunit (Rpb1) carboxy-terminal domain (CTD) containing heptad repeats with the consensus sequence Tyr1-Ser2-Pro3-Thr4-Ser5-Pro6-Ser7. Consensus repeats have five potential phosphorylation sites. Amino acid residues that differ from the consensus motif are depicted in red. (**b**) Treatment of HeLa cells with nocodazole induced a slower migrating Thr4-P-specific Pol II00 form. Cells were treated with nocodazole (20 ng/ml) for the indicated time points and extracts of adherent and shake off cells were analyzed by western blotting with mAbs specific for CTD modifications Thr4-P (6D7), Ser2-P (3E10), Ser5-P (3E8), Ser7-P (4E12), or Rpb1 (Pol 3.3). II0 and IIA represent the known hyper- and hypophosphorylated forms of the large subunit Rpb1 of Pol II; II00 represents a new slower migrating form. H3Ser10-P served as a marker for mitotic cells and actin was the loading control. WCE, whole cell extract. a, asynchronous cells. m, mitotic shake off cells. (**c**) Treatment of HeLa cells with 500 nM okadaic acid (OA) induced the Thr4-P-specific Pol II00 form. Cells were treated with different concentrations of OA for 90 min and whole cell extracts were analyzed by western blotting with antibodies that detected specific CTD modifications, Rpb1 or H3Ser10-P. Tubulin served as the loading control.

The CTD of transcriptionally engaged Pol II is phosphorylated at Tyr1, Ser2, Thr4, Ser5, and Ser7 residues by various kinases in interphase cells^21^ (Fig. 1a). At the beginning of M phase Pol II dissociates from chromatin and distributes evenly in the cytoplasm. To compare the phosphorylation state of Pol II in interphase and mitotic cells we arrested HeLa cells in pro-metaphase with nocodazole. Nocodazole prevents the polymerization and attachment of microtubules to kinetochores and activates the spindle assembly checkpoint. Extracts were prepared from detached and adherent cells at different time points after addition of nocodazole and studied with CTD phosphorylation specific antibodies by Western blots. Nocodazole induced a shift of the transcriptionally engaged Pol II0 to the transcriptionally inactive Pol IIA form four hours after treatment (Fig. 1b, lane 3, αRpb1). Consistent with this shift the signals for CTD Ser2-P and Ser5-P were strongly reduced. In contrast, Ser7-P signals declined only moderately at 4 hour and recovered thereafter. Strikingly, a new form of Pol II was detected in extracts of nocodazole-treated cells. This form, which we called II00, migrates significantly slower than the II0 form in gel electrophoresis, reacts strongly with a Thr4-P-specific antibody, but does not react with Ser2-P, Ser5-P, and Ser7-P-specific antibodies. The new II00 form was barely detectable with the Rpb1-specific mAb Pol3.3, which recognizes an epitope outside of the CTD in Rpb1. We conclude that the Pol II00 form is little abundant in M phase cells and represents less than 10% of total Pol II. The Pol II00 form was detectable also in nocodazole-treated HepG2 and H1299 cells (Supplementary Fig. 1a) and with other Thr4-P-specific mAbs of different subclass (Supplementary Fig. 1b–e).

The phosphatase inhibitor okadaic acid can induce a cell cycle arrest in interphase cells accompanied by chromatin condensation, nuclear lamina depolymerization, and partial separation of centrosomes^34^,^35^. Okadaic acid also induces, similar to nocodazole, phosphorylation of histone H3 Ser10 residues and leads to activation of Polo-like kinase 1 (Plk1), both hallmarks of M phase entry. Therefore we asked, whether okadaic acid can also induce the mitosis-specific, Thr4 phosphorylated form of Pol II CTD. HeLa cells were treated with increasing concentrations of okadaic acid for 90 min and the different CTD modifications were analyzed in whole cell extract by Western blot. While okadaic acid did not affect the levels of Ser2, Ser5, and Ser7 phosphorylation of the Pol II0 form, high concentrations of okadaic acid induced the Thr4 phosphorylated Pol II00 form. Again, the II00 form showed no signs of Ser2, Ser5, or Ser7 phosphorylation (Fig. 1c). We conclude that okadaic acid induces, in addition to previously reported mitosis-specific markers, also the Pol II00 form. The exclusive phosphorylation of Thr4 residues in the Pol II00 form may involve either the activation of a mitosis-specific kinase or the inhibition of a Thr4-P-specific phosphatase. The possible function of the mitosis-specific Plk1 for Thr4 phosphorylation will be addressed further below.

### Thr4 phosphorylated Pol II associates specifically with centrosomes and midbody

**Figure 2:**
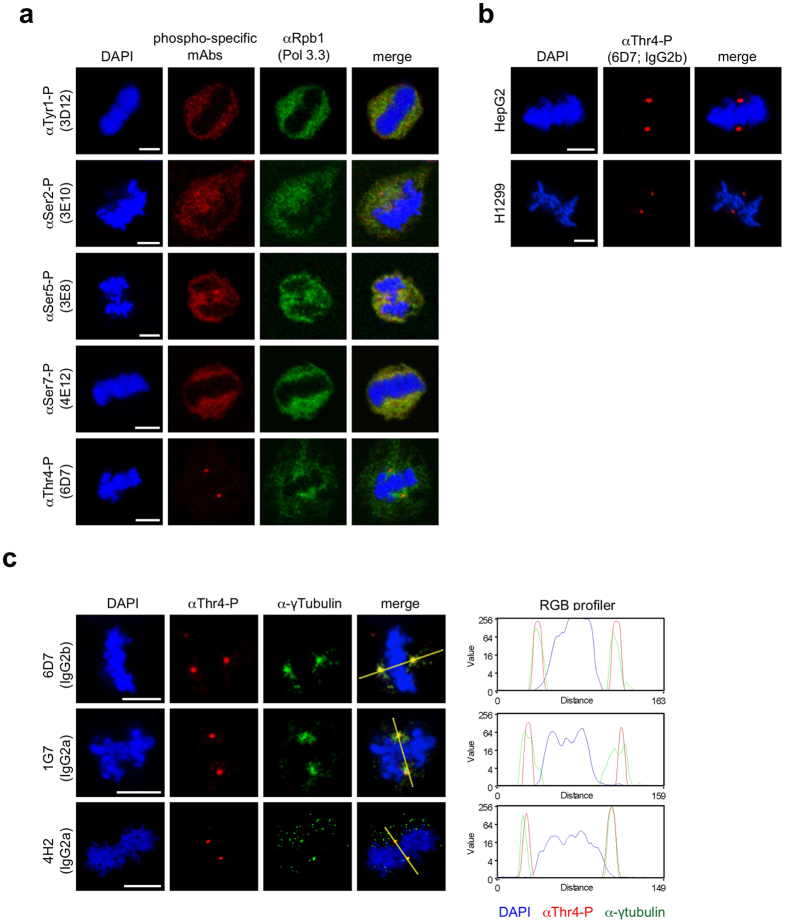
Distribution of Pol II populations during mitosis. (**a**) The Pol 3.3 monoclonal antibody (mAb) (αRpb1) recognizes a Pol II epitope outside of the CTD and allows the visualization of the distribution of total Pol II in cells (green). Tyr1-P, Ser2-P, Ser5-P, Ser7-P, or Thr4-P-specific mAbs show the abundance and distribution of individual CTD modifications (red) during mitosis. Merged images show localization of signals in the cytoplasm, but also reveal strong enrichment of Thr4-P signals in two distinct foci (red). DAPI: 4′,6-diamidino-2-phenylindole. Representative images of metaphase chromosomes are shown. Data are from three experiments in which at least 100 cells were analyzed and >98% of mitotic cells show these Pol II CTD-P distributions. (**b**) Images of a Thr4-P-specific mAb (6D7) and DNA (DAPI) in different mitotic human cells (HepG2, H1299). (**c**) Immunofluorescence images of Thr4-P-specific mAbs of different subclasses (6D7, 1G7, 4H2; red) and a centrosome-specific Ab (γ-tubulin; green) in HeLa cells. Representative images of metaphase chromosomes from three experiments are shown. Signals were merged and quantified using Image J 1.37 V and the plug-in RGB profiler. Line scans were used to measure Thr4-P-specific and γ-tubulin-specific signals. Scale bars, 5 μm.

At the beginning of mitosis the large majority of Pol II dissociates from chromatin and localizes dispersed in the cytoplasm of mitotic cells (Fig. 2aαRpb1). Apparently, this dissociation does not result in a complete dephosphorylation of the CTD. Considerable amounts of Tyr1-P, Ser2-P, Ser5-P, and Ser7-P modified CTD are detectable in the cytoplasm of mitotic cells (Supplementary Fig. 2). Notably, the signals for Thr4-P diverged significantly from the distribution of other CTD modifications and were strongly enriched at two distinct foci in mitotic HeLa cells (Fig. 2a). The specific enrichment of Thr4-P signals was seen also in mitotic HepG2 and H1299 cells (Fig. 2b), and if other Thr4-P-specific monoclonal antibodies of other subclasses were applied (Fig. 2c). We wondered, whether the two Thr4-P positive spots seen in mitotic cells may correspond to centrosomes since they were symmetrically arranged relative to the condensed chromatin. In fact, the co-localization of Thr4 phosphorylated Pol II with centrosomes was confirmed after co-staining with a γ-tubulin-specific antibody. The merge of γ-tubulin and Thr4-P signals revealed the Thr4-P signals in the central region rather than in the periphery of centrosomes, as indicated by the RGB profiler (Fig. 2c).

We next asked whether the association of Thr4-P signals with centrosomes is seen in all phases of mitosis and whether the association with centrosomes can be seen also in other cell cycle phases. Inspection of mitotic cells detected the Thr4-P signal consistently at centrosomes in prophase, metaphase, anaphase, and telophase cells (Supplementary Fig. 3). A strong enrichment of the Thr4-P signal was further observed for the midbody of telophase cells (Supplementary Fig. 3a, white arrow). Importantly, the inspection of a large panel of cells never revealed the association of Thr4-P signals with centrosome(s) of interphase cells (Supplementary Fig. 3a). Therefore, we conclude that the Thr4 phosphorylated form of Pol II associates specifically with centrosomes and the midbody of M phase cells.

### Recruitment of Pol II to centrosomes does not require the CTD

**Figure 3:**
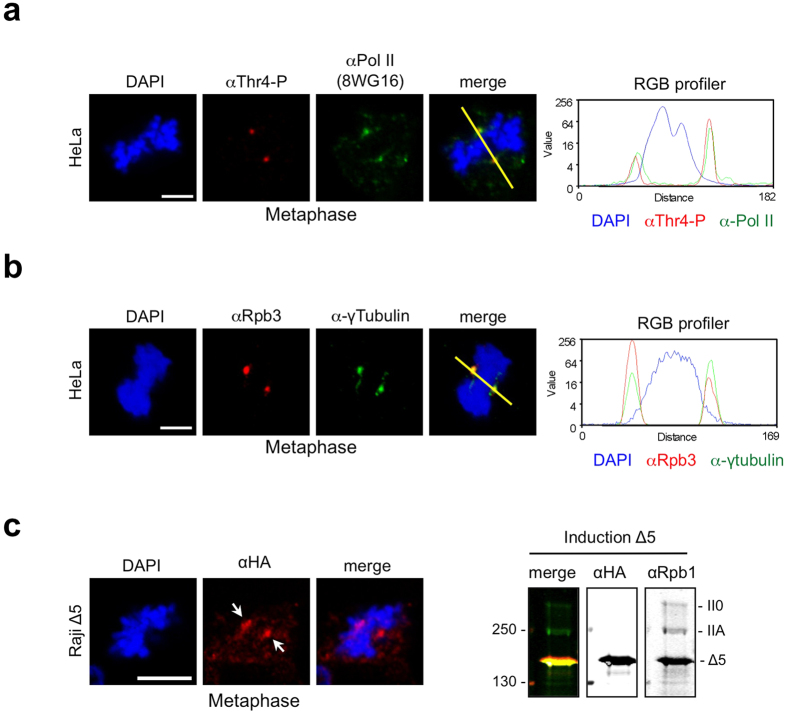
Thr4 phosphorylated Pol II co-localizes with centrosomes in M phase. Representative images of a metaphase cells treated with 0.1% Triton X-100 before PFA fixation. (**a**) HeLa cells were stained with a Thr4-P-specific mAb (6D7, red) and mAb 8WG16 (green) recognizing the CTD of Pol II independently of specific modifications. Co-localisation of Thr4-P and 8WG16 mAbs signals was detected in >98% of mitotic cells. (**b**) Immunofluorescence image of Rpb3 mAb recognizing the human RNA polymerase II subunit 3 (red) and a centrosome-specific Ab (γ-tubulin; green). Signals from merged images were quantified using Image J 1.37 V and the plug-in RGB profiler. Line scans were used to measure the relative localizations of 8WG16/Rpb3 and Thr4-P-specific signals. (**c**) Conditional expression of Δ5 mutant in Raji cells. Recombinant polymerase were induced by removal of tetracycline. After 24 h the expression levels of endogenous and recombinant polymerases were analyzed by western blot. The HA mAb allows the visualization of recombinant HA-tagged Pol II (red). Recruitment of Pol II to centrosomes occurs independently of the CTD. Scale bars, 5 μm.

Next we studied the recruitment of Pol II to centrosomes. We asked, whether a fully assembled Pol II enzyme interacts with centrosomes and, whether the CTD is required for the interaction. Mitotic cells were permeabilized and washed with a buffer containing the non-ionic detergent Triton X-100. Under these conditions Thr4-P signals remain stably associated with centrosomes (Fig. 3a), while the fraction of Pol II phosphorylated at Tyr1, Ser2, Ser5, and Ser7 residues is largely removed from the cytoplasm (data not shown). To corroborate the evidence for binding of fully assembled Pol II to centrosomes we tested further Pol II-specific mAbs. The mAb 8WG16 recognizing the CTD of Pol II in a phosphorylation independent manner and stains two prominent spots in mitotic HeLa cells, which matches the Thr4-P-specific spots (Fig. 3a), suggesting that 8WG16 and Thr4-P-specific mAbs recognize the same cellular structure. These two spots are further recognized by αRpb3, a Pol II subunit 3-specific antibody, and a γ-tubulin-specific antibody (Fig. 3b, Supplementary Fig. 3b). Thus, antibodies for different subunits recognize Pol II at centrosomes in mitotic cells. Therefore, we conclude that a fully assembled Pol II is recruited to centrosomes.

Is the CTD required for Pol II recruitment to centrosomes? To answer this question, we expressed and analyzed a HA-tagged version of Pol II lacking the CTD (Δ5) in human B cell line Raji. Previous studies showed that the deletion of CTD does not interfere with the proper assembly of Pol II *in vivo*. However, Δ5 has a reduced transcriptional activity on reporter gene constructs^36^,^37^ and is transcriptionally dead on chromatin-packaged genes^37^. Similar to wild type Pol II the CTD-truncated version Δ5 is consistently enriched at centrosomes when a large panel of mitotic cells was analyzed (Fig. 3c). This demonstrates that recruitment of Pol II can occur independently of the CTD.

### Interaction of centrosome-specific factors with Pol II

**Figure 4:**
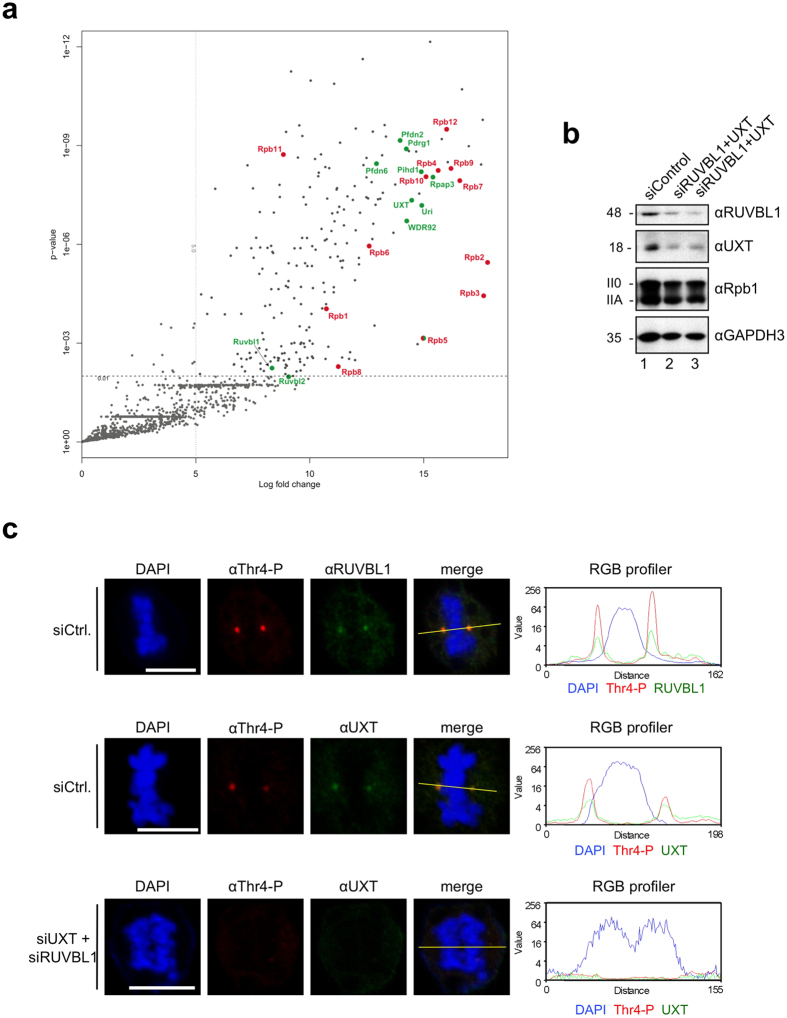
Knockdown of Pol II interactors RUVBL1 and UXT abolishes Thr4-P levels at centrosomes. (**a**) The interactome of Pol II. Volcano plot displaying the significant proteins interacting with the recombinant Rpb1 (αHA) when compared to the controls (Pes1 and Bop1). Thresholds: Log 2 Fold change ≥ 5; p-value < 0.01. Indicated are the 12 subunits of Pol II subunits (red) and the 11 subunits of the non-canonical R2TP-Prefoldin-like complex (green). Data based on three biological independent replicates. (**b**) Western blot analysis of extracts from HeLa cells 48 h after siRNA knockdown of RUVBL1 and UXT. GAPDH3 served as a loading control. (**c**) Signals for Thr4-P (6D7, red) as well as RUVBL1/UXT (green) co-localized in each phase of mitosis. Representative images of metaphase and anaphase chromosomes are shown. Immunofluorescence images of UXT (green) and Thr4-P-specific (red) mAb in HeLa cells 48 h after siRNA knockdown. >30 cells were analyzed for each experiment; 13% of mitotic cells show loss of the signal for UXT (or RUVBL1) at centrosomes accompanied by the loss of the signal for Thr4-P. Line scans were used to measure the relative localizations of RUVBL1, UXT and Thr4-P-specific signals. Scale bars, 5 μm.

The stable association of Pol II with centrosomes during mitosis has not been described before by mass spectrometry analysis or other techniques. A possible explanation for this lack of evidence could be that the interaction of Pol II with centrosomes in mitotic cells is sensitive to specific purification steps and disrupted when cellular extracts are prepared. Therefore we asked whether the interactomes of Pol II and centrosomes share common factors and whether these factors can give a clue how Pol II is recruited to centrosomes. Our mass spectrometric analysis of the Pol II interactome (see Methods) revealed several large complexes as the Mediator, Integrator, and Proteasome as well as many smaller complexes and single factors. Our particular attention gained the non-canonical R2TP-Prefoldin-like complex composed of PFDN2, PFDN6, UXT, PDRG1, URI, Rpb5, WDR92, RPAP3, PIHD1, RUVBL1 and RUVBL2^38^ (Fig. 4a). This complex is not only required for assembly of RNA polymerases in the cytoplasm^39^, but is essential also for the assembly of microtubules and the spindle apparatus in mitotic cells^40^. Several subunits of the non-canonical R2TP-Prefoldin-like complex including RUVBL1, RUVBL2^41^,^42^, and UXT^43^ have been described as centrosome constituents and are associated with Pol II in mitotic cells^44^. A potential functional link between Pol II and centrosomes is further supported by a recent global protein interaction study in mammalian cells. In this study, several hundreds so-called communities of protein interactions were defined as well as the interaction between these communities^45^. Interestingly, a community of factors with centrosomal functions showed by far the highest functional interaction with the community of kinetochore specific factors and the community containing the subunits of RNA polymerases^45^. Therefore we asked whether the knockdown of components of the non-canonical R2TP-Prefoldin-like complex affects the association of Pol II with centrosomes in mitotic cells. For this purpose, subunits UXT and RUVBL1 were knocked down to 10–20% of control cells without having a significant impact on the levels and ratio of Pol IIA and Pol II0 (Fig. 4b). The knockdown did not prevent entry of cells in mitosis and the formation of metaphase, anaphase, and telophase cells suggesting that the spindle apparatus and centrosomes are in principle functional in mitotic cells (Fig. 4c, DAPI stain). However, the loss of the signal for UXT (or RUVBL1) at centrosomes was consistently accompanied by the loss of the signal for Thr4-P (Fig. 4c, Supplementary Fig. 4). We can currently not discriminate whether the absence of Thr4-P signals at centrosomes in mitotic cells is solely due the disturbed recruitment of Pol II, or whether a more severe damage of centrosomes occurs after UXT/RUVBL1 double knockdown preventing Pol II recruitment.

### Plk1 associates with Pol II in mitotic cells and is a Thr4-specific kinase

**Figure 5:**
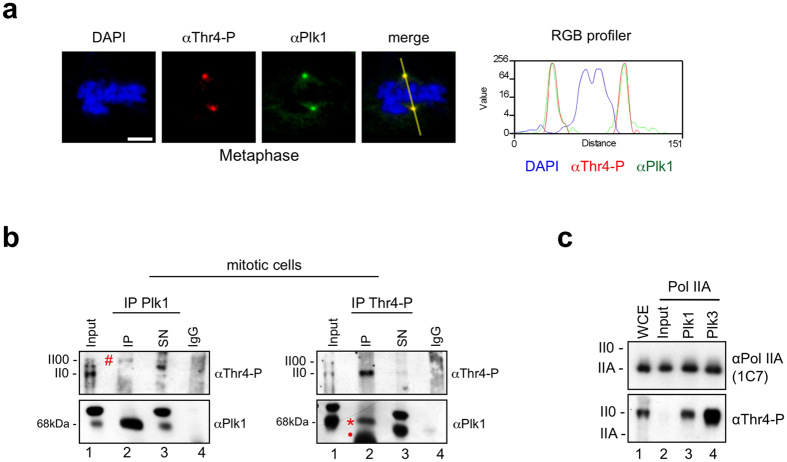
Plk1 and Thr4-P modified Pol II interact *in vivo* and co-localize at centrosomes in M phase cells. (**a**) Co-staining of Plk1 (green) and Thr4-P (6D7; red) in HeLa cells. Representative image of metaphase chromosomes is shown. Line scans measured the relative localization of Plk1 and the Thr4-P-specific signals. Scale bars, 5 μm. (**b**) HeLa cells were synchronized with nocodazole (20 ng/ml) for 8 h and mitotic cells were collected using the shake off technique. Immunoprecipitation (IP) experiments with antibodies against Plk1 and CTD-Thr4-P (6D7) from extracts of shake off HeLa cells. Immunoprecipitates were analyzed by western blotting with the indicated antibodies. SN, supernatant; IgG, rabbit serum, isotype control. Asterisks, hash-tags and dots indicate bands for Plk1, Thr4-P and heavy chain, respectively. (**c**) Immunoprecipitated Pol IIA from HeLa cell extracts was used as a substrate for Plk1 and Plk3, and analyzed by Western blotting with mAbs specific for Thr4-P or non-modified CTD (1C7). WCE, whole cell extract.

Modification of Pol II CTD at centrosomes is characterized by the absence of phosphorylation of Tyr1, Ser2, Ser5, and Ser7 and the strong phosphorylation of Thr4 residues, while Pol II in the cytoplasm of mitotic cells is positive for all five phosphorylation marks. It is currently unclear how the specific phosphorylation of Pol II at centrosomes is achieved. Pol II from the cytoplasm could be recruited to centrosomes and Tyr1-P, Ser2-P, Ser5-P, and Ser7-P marks are removed by centrosome-associated phosphatases except Thr4-P. Alternatively, removal of all CTD phosphorylation marks occurs first and subsequently Thr4 gets specifically phosphorylated at centrosomes. Recently we describe the phosphorylation of CTD Thr4 residues in interphase cells by Polo-like kinase 3 (Plk3)^6^. Plk3 is expressed in all phases of the cell cycle, while expression of other Plk family members is restricted to M phase, where they play a pivotal role in the regulation of M phase progression. Plk1, the major constituent of the Plk family, is associated with centrosomes and the midbody during mitosis (Fig. 5a, Supplementary Fig. 5a). To examine the possible interaction of Pol II with Plk1 in mitotic cells, we performed co-IP experiments. Plk1-specific mAbs co-precipitated a small fraction of the Pol II00 form of nocodazole-treated cells (Fig. 5b, hashtag), and vice versa a Thr4-P-specific mAb precipitated Plk1 (Fig. 5b, asterisks). The association of Pol II and Plk1 was not seen after immunoprecipitation of Pol II with Ser2-P, Ser5-P, and Ser7-P-specific mAbs (data not shown). We next examined, if Plk1 is a CTD Thr4-specific kinase. The hypophosphorylated Pol IIA form was immunoprecipitated and purified with mAb 1C7, which recognizes only the unmodified CTD. Recombinant Plk1 (Proqinase Freiburg) phosphorylated Pol IIA specifically at Thr4 residues and caused a shift to the Pol II0 form (Fig. 5c), similarly as described before for Plk3^6^. Plk1 and Plk3 did not phosphorylate CTD residues Ser2, Ser5, or Ser7 (data not shown). Notably, *in vitro* phosphorylation of CTD Thr4 residues by Plk1 and Plk3 did not shift the Pol IIA to the II00 form. We assume that further, currently unknown modifications contribute to the shift of Thr4 phosphorylated Pol II0 to the II00 form *in vivo*.

We further performed knockdown experiments for Plk1 in various cell lines to ask, if Plk1 is responsible for the Thr4 phosphorylation in mitotic cells. The knockdown of Plk1 (Supplementary Fig. 5b) turned out to be highly toxic and the large majority of cells were detached from cell dishes 24 h after transfection of Plk1-specific siRNAs. The remaining adherent cells were fixed and prometaphase and metaphase cells were microscopically analyzed. In these cells the loss of Plk1 signals at centrosomes was consistently accompanied by the loss of the Thr4-P signal (Supplementary Fig. 5c). Again, we cannot rule out that the absence of Thr4-P signals at centrosomes after knockdown of Plk1 is caused by problems of proper centrosome formation and Pol II recruitment to centrosomes. However, all together our data support the notion that Plk1 is a Pol II interacting Thr4-specific CTD kinase.

### CTD Thr4 mutants inhibit proper M phase progression

**Figure 6:**
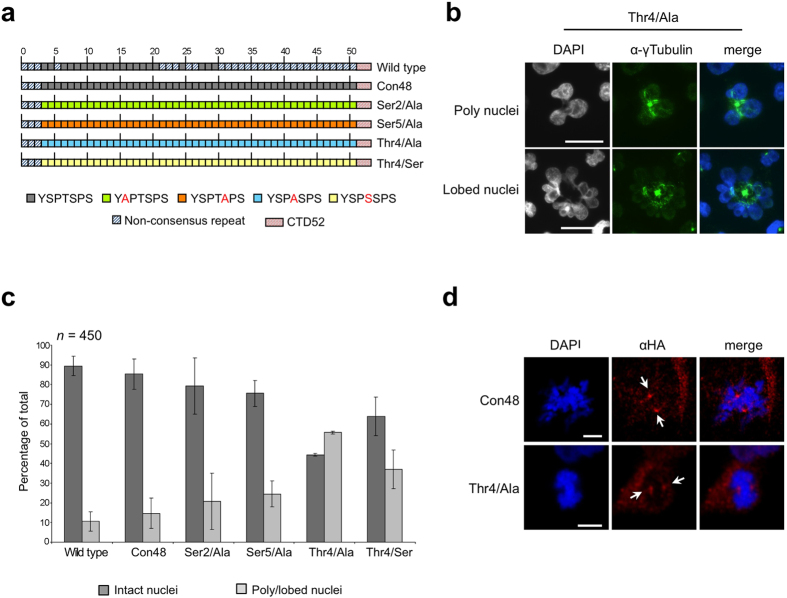
CTD Thr4 mutants inhibit proper M phase progression. The phenotype of CTD mutants was studied in human B cell line Raji after expression of CTD mutants. Removal of tetracycline induced expression of (**a**) recombinant Rpb1 CTD mutants. (**b**) Immunofluorescence images of γ-tubulin (green) and DNA (DAPI) signals 24 h after induction. (**c**) Percentage of normal and abnormal cells, with polyploid or lobed nuclei was determined. Data are from three experiments; *n* = 450, number of cells analyzed for each mutant. Error bars represent standard deviations. (**d**) The HA mAb allows the visualization of the distribution of recombinant Pol II in cells (red). Arrows indicate a strong enrichment of Pol II in two distinct foci symmetrically to condensed chromatin in metaphase cells. Scale bars, 5 μm.

The binding of a specifically Thr4 phosphorylated Pol II at centrosomes in mitotic cells is striking. We asked therefore, whether Thr4 residues in CTD heptad repeats might have a mitosis-specific function required for progression of cells through M phase. To answer this question, we studied a panel of Pol II mutants in a conditionally inducible Raji B cell system. Pol II mutants have either replaced Ser2, Thr4, or Ser5 residues by alanine, or Thr4 by serine in heptad repeats 4–51 (Fig. 6a). Mutant Con48 containing consensus repeats served as control^6^. Expression of Pol II mutants was induced for 24 h. Cells were washed, collected on glass slides by cytospin centrifugation, fixed, and stained with DAPI, and γ-tubulin and Thr4-P-specific mAbs. Subsequently, cells were carefully inspected for cells having passed an abnormal mitosis or cytokinesis.

In our analysis we distinguished three categories of cells: (I) normal cells, with a single, regularly shaped nucleus, and abnormal cells with either (II) two or more nuclei (poly nuclei), or (III) cells with strongly lobed nuclei (lobed nuclei) (Fig. 6b). The phenotypes II and III are associated with M phase defects. Wild type Raji cells expressing only endogenous Pol II displayed ~10% abnormal cells of categories II and III. The proportion of abnormal cells increased to ~15% after expression of the mutant Con48 and to ~20% and ~25% after expression of the mutants Ser2/Ala or Ser5/Ala, respectively. The highest proportion of abnormal cells was detected after expression of the mutants Thr4/Ser (~39%) and Thr4/Ala (~55%) (Fig. 6c, Supplementary Fig. 6). All recombinant polymerases carry a point mutation conferring resistance to α-amanitin and their expression, except Con48, cannot maintain viability of cells in the presence of α-amanitin^6^. The experiments here were performed in the absence of α-amanitin and the observed phenotype is induced by overexpression and binding of mutant Pol II to centrosomes (Fig. 6d). Expression of the mutants Thr4/Ala and Thr4/Ser caused by far the most severe phenotype in M phase cells and affected mitosis as well as cytokinesis of cells. Because Ser2/Ala, Ser5/Ala, Thr4/Ala, and Thr4/Ser mutants all display a severe global phenotype on gene transcription in interphase cells, if cells are cultured in the presence of α-amanitin^3^,^6^ we assume that the M phase-specific phenotype of Thr4/Ala and Thr4/Ser mutants is not caused by a transcriptional defect of these mutants. It is more likely that the M phase-specific phenotype is caused by the lack of Thr4 phosphorylation of CTD after recruitment of Pol II to centrosomes.

## Discussion

The study of phosphorylation states of CTD in mammalian M phase cells led to the identification of a Thr4 phosphorylated Pol II form, which is exclusively associated with centrosomes and midbody. To date, the currently known function of Pol II in M phase cells is restricted to transcription of telomeric and centromeric sequences. Transcription of these sequences is associated with the Ser2 and Ser5 phosphorylated form of Pol II CTD and essential for proper kinetochore function^30^,^31^.

The finding of a specifically Thr4-P modified form of Pol II at centrosomes and midbody of M phase cells is striking. The physical presence of Pol II at centrosomes and midbody was corroborated with various Pol II-specific mAbs. Signals were detectable for three different mAbs with specificity for Thr4-P, for mAb 8WG16 recognizing unmodified CTD, and for a mAb specific for subunit Rpb3 of Pol II. Binding of Pol II to centrosomes during mitosis was further corroborated with HA-tagged recombinant Rpb1. The CTD of Pol II at centrosomes and midbody was essentially not phosphorylated at Tyr1, Ser2, Ser5, and Ser7 residues, which are characteristic modifications of transcriptionally engaged Pol II in interphase cells. A second hallmark of M phase cells is the new high molecular weight form of Pol II, Pol II00, which is phosphorylated at Thr4 residues, but not at other CTD phosphoacceptor sites. Even though it is very likely that the Pol II00 form is the subfraction associating with centrosomes and midbody in M phase cells a direct proof for this assumption is still missing. Also the cause for the molecular shift of the Pol II00 form and whether the reason for the shift involves modification of the CTD or other parts of Rpb1 is yet unclear. Importantly, the CTD is not required for anchoring Pol II at centrosomes. This is consistent with the assumption that subunits of the non-canonical R2TP-Prefoldin-like complex could be involved in recruitment and/or tethering of Pol II to centrosomes. The molecular mechanism underlying the recruitment of Pol II to centrosome requires further analyses in the future.

If centrosomes and the midbody do not contain template DNA for transcription, the question raises: what is the molecular purpose of an exclusively Thr4 phosphorylated Pol II at centrosomes and midbody? The CTD is composed mainly of Tyr, Ser, Thr, and Pro residues and, on the basis of its amino acid composition, belongs to the family of low complexity (LC) protein domains. Founding members of the family of LC proteins include Fused in Sarcoma (FUS), Ewing Sarcoma (EWS), and Basel Transcription Factor 15 (TAF15), which all harbor domains with multiple repetitions of the amino acid motif (S/G) Y (S/G)^46^. Proteins with LC domains can form stable fibrillous networks and undergo liquid-liquid phase transition. The LC domains remain unstructured in fibrillous networks and are probably stabilized by multiple weak interactions of Tyr residues. The phenomenon of liquid-liquid phase separation of LC domain containing proteins has attracted high attention because it can explain the transient and stable formation of cellular granules. Several recent publications have provided evidence that the CTD of Pol II can form aggregates with other LC proteins in hydrogels and in liquid droplets and that this aggregation is controlled by phosphorylation of CTD^47^,^48^,^49^.

**Figure 7:**
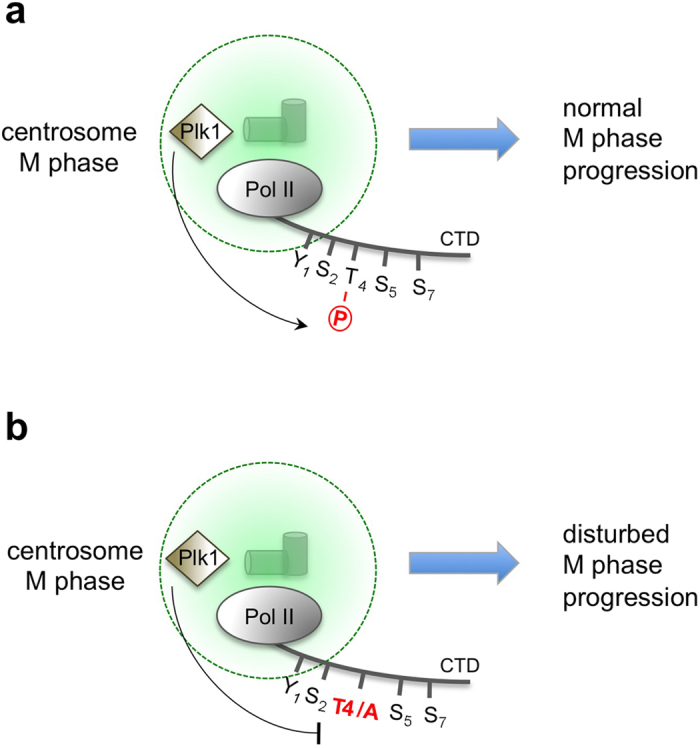
Model of Pol II function in M phase. (**a**) Pol II is recruited to centrosomes of mitotic cells and subsequently phosphorylated at CTD Thr4 residues by Plk1 to promote proper M phase progression. (**b**) Recruitment of Pol II Thr4/Ala mutant to centrosomes disturbs M phase progression.

There is also evidence now that proteins with LC domains have specific functions in mitosis. The evolutionary conserved LC protein BuGZ is required for kinetochore function and chromosome alignment^50^. BuGZ undergoes phase transition to promote assembly of both mitotic spindles and their associated components^51^. The composition and structural nature of the spindle matrix of mitotic cells is currently little defined, but there is increasing evidence that a spindle matrix constitutes a real structure of a defined network of proteins. A common feature of spindle matrix associated proteins is that they retain their local integrity upon disassembly of microtubules^51^,^52^,^53^,^54^. Assuming that a spindle matrix is established in mitotic cells what could be the starting point(s) for its building? Centromeres and centrosomes are both central structures of mitotic cells regulating separation and proper segregation of chromosomes. Binding of Pol II to centrosomes may play a critical function in this process. Pol II can bind to centrosomes possibly with the help of subunits of the non-canonical R2TP-Prefoldin-like complex. A recent detail mapping of close protein neighborships for the human centrosome proteins using the proximity-dependent biotinylation method revealed a cluster of centrosome peripheral proteins, termed Tz1, containing multiple subunits of the non-canonical R2TP-Prefoldin-like complex^55^. It is tempting to speculate that Pol II is recruited to this specific cluster of centrosomal proteins, which functionally coordinates and regulates the growth of microtubules. At centrosomes CTD could be specifically phosphorylated at Thr4 residues by Plk1 and this structure may contribute to the formation of a stable spindle matrix (Fig. 7). The concentration of Pol II phosphorylated at CTD Thr4 residues may function as an initialization point for priming the interaction of CTD with other LC domain proteins, which help shaping the spindle apparatus in mitotic cells.

This model is supported by the observation that Thr4/Ala and Thr4/Ser mutants display a severe mitosis defect. Both mutants may fail to build and maintain an appropriate spindle apparatus. In principle, Pol II CTD Thr4 mutants could cause M phase defects also by affecting proper expression of mitosis-specific genes during interphase. However, this scenario appears unlikely because the exclusive phosphorylation of Thr4 residues in CTD occurs only in M phase cells and is linked to the physical association of Pol II to centrosomes and midbody. Taken together, our results describe an essential function of CTD for progression of cells through M phase, which most likely is not coupled to the transcriptional activity of Pol II. Whether CTD serves as platform to facilitate the aggregation with other proteins, e.g. LC proteins, and thereby supports the establishment of a spindle matrix is currently unknown and deserves further investigations.

## Methods

### Cell Lines

HeLa, H1299 and HepG2 cells were incubated in DMEM medium supplemented with 10% foetal calf serum (FCS), 100 U/ml penicillin, 100 μg/ml streptomycin and 2 mM L-glutamine (DMEM, Gibco) at 37 °C and 8% CO_2_. Raji cells were grown in RPMI 1640 medium supplemented with 10% FCS, 100 U/ml penicillin, 100 μg/ml streptomycin and 2 mM L-glutamine (Gibco/Invitrogen) at 37 °C and 5% CO_2_. Stable-transfected Raji cell lines were produced and maintained by selection with G418 (Sigma)^3^,^56^. Removal of tetracycline induced cell lines to express recombinant Rpb1.

### Antibodies

Monoclonal antibodies (mAbs) specific for Rpb1 (Pol 3.3), non-modified CTD (1C7), haemagglutinin (HA)-tag (3F10, Roche), Pol II CTD (8WG16) and the different CTD phosphorylations (3E10, 3E8, 4E12, 6D7, 1G7, 3D12) were used as described previously^3^,^4^,^6^. The rat mAb 4H2 (IgG2a) against CTD-Thr4-P was generated as described previously^3^. For immunization, we used CTD-specific Thr4-specific phosphopeptides (YSPT^P^SPSYSPTSPSC) coupled to ovalbumin (Peptide Specialty Laboratories GmbH, Heidelberg, Germany). The WDR43 (1B8; IgG2b) (unpublished data), Pes1 (pescadillo1; 8E9; IgG1) and Nog1 (1D8; IgG2a) specific antibodies are isotype controls described elsewhere^57^,^58^. Affinity purified antibodies used in this paper were as follows: Rpb3 (Bethyl Laboratories; A303–771A), Plk1 (Abcam; ab47867), γ-tubulin (Sigma; T6557), α-tubulin (Sigma; T6199), β-actin (Sigma; A2066), normal rabbit IgG (Santa Cruz; sc-2027), H3Ser10P (Active Motif; 39253), RUVBL1 (Proteintech; 10210–2-AP) and UXT (Abcam; ab77483).

### Cloning of CTD mutants

Recombinant CTD constructs were created essentially as previously described^56^, and transferred to a tetracycline-regulated expression vector. In brief, pUC19-CTD modified to contain an *Nhe* I site in repeat 3 was digested with *Nhe* I and *Sty* I, to which synthetic linkers with the sequence for three heptads with e.g. Ser7/Ala mutations, were unidirectionally introduced. Isoschizomers of *Nhe* I (*Avr* II and *Spe* I) included in the linkers permitted sequential cloning of multimers from (*Nhe* I - *Cla* I) back into the same vector (*Spe* I - *Cla* I), leading to a doubling of repeats at each step. Joining of *Spe* I to *Nhe* I encodes Thr-Ser, allowing seamless joining and expansion of repeat multimers. All generated constructs were sequenced.

### Immunofluorescence microscopy

HeLa, H1299 and HepG2 cells were grown on a coverslip for 24 h with DMEM/10% FCS complete medium. For application of the 8WG16 mAb and the Rpb3 polyclonal antibody, the cells were first permeabilized with 0.1% TritonX-100 for 15 min at room temperature (RT), washed 5 min with PBS and fixed with 3.7% or 2% paraformaldehyde (PFA) at RT for 5 min, respectively. For all other antibodies, the cells were first fixed with 2% PFA at RT for 5 min and subsequently permeabilized with 0.15% TritonX-100 for 15 min at RT. All samples were blocked with 1% BSA for 30 min and incubated with the appropriate primary antibody over night at 4 °C. Cells were washed with PBS, 0.15% TritonX-100 for 10 min at RT, blocked with 1% BSA for 7 min and incubated with Cy3-conjugated goat anti-rat immunoglobulin (Dianova) and Alexa Fluor 488 goat anti-mouse immunoglobulin (Molecular Probes) in the dark for 45 min. Cells were washed again with PBS containing 0.15% TritonX-100, stained with 4′,6-diamidino-2-phenylindole (DAPI) (Sigma), and mounted on slides using fluorescent mounting medium (Dako). Confocal microscopy was performed on a Leica LSCM SP2 fluorescence microscope. Images were taken with objective HCX PL APO 63 × 1.4 and processed using Image J 1.37 V software and the plug-in RGB profiler. Fluorescence microscopy was performed on an Axiovert 200 M microscope. Scale bars were calculated as follows:


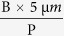


B = picture length in μm

P = (512 pixel × voxel size) in μm

If not stated otherwise in the figure legends, in each experiment >30 cells were analyzed.

### Immunoprecipitation (IP)

Enrichment of mitotic cells was performed as follows: HeLa cells (70% confluency) were synchronized with nocodazole (Sigma; 20 ng/ml) for 8 h and mitotic cells were collected by shake off. HeLa cells (4 × 10^6^) were lysed in 200 μl IP buffer (50 mM Tris-HCl, pH 8.0, 150 mM NaCl, 1% NP-40 (Roche), 1× PhosSTOP (Roche), 1× protease inhibitor cocktail (Roche) for 20 min on ice. All samples were sonicated on ice using a BRANSON Sonifier 250 (15 sec on, 15 sec off, 50% duty) and centrifuged at 14.500 rpm for 15 min at 4 °C. The supernatant was incubated with antibody-coupled protein G/A-sepharose (1:1) beads (2.5 μg of antibodies for 4 h at 4 °C, followed by two washes with 1 ml IP buffer) with rotation, overnight. Beads were washed six times with 1 ml IP buffer and were used as substrate for *in vitro* kinase assay or the proteins were boiled off the sepharose beads in Laemmli buffer, containing 8 M urea, for sodium dodecyl sulphate-polyacrylamide gel electrophoresis (SDS-PAGE). For optimizing the IP efficiency of the 6D7 antibody against CTD-Thr4-P, G/A-sepharose beads were preincubated with a bridging antibody (Dianova; AffiniPure anti-rat) for 1 h at RT before primary antibody binding.

### *In vitro* kinase assay

Endogenous Pol II was immunopurified from HeLa whole cell extracts using an antibody recognizing non-modified CTD (1C7). 10 μl of the substrate-coupled protein Sepharose G beads were incubated with 40 μl kinase buffer B (50 mM Hepes (pH 7.9), 100 mM KCl, 10 mM MgCl_2_, 200 μM EGTA, 100 μM EDTA, 1 mM DTT, 200 μM ATP, 1 μg BSA and 200 ng of the recombinant kinase) at 30 °C for 30 min. Specific kinase activity specified by the supplier (Proqinase, Freiburg) in pmol/μg × min were: Plk3 (131), Plk1 (152). Laemmli buffer was added (6-fold) and samples were incubated for 5 min at 95 °C followed by western blot analysis.

### RNA interference

Synthetic short interfering RNA (siRNA) oligonucleotides (Eurofins/MWG) were transfected into cells using Oligofectamine (Invitrogen). HeLa cells were seeded at a density of 100,000 cells/well in 6-well plates the day before transfection. SiRNA oligonucleotide (100 nM) diluted in 150 μl Opti-MEM (Invitrogen) was incubated with 3 μl Oligofectamine (Invitrogen) for 25 min at RT and then mixed with 600 μl Opti-MEM. The transfection mixture was added to cells and incubated for 6 h. The following siRNA sequences were used:

Luciferase: 5′-CGUACGCGGAAUACUUCGAdTdT-3′;

human Plk1: 5′-AGACCUACCUCCGGAUCAAdTdT-3′;

human RUVBL1: 5′-UGGCGUCAUAGUAGAAUUAAUdTdT-3′;

human UXT: 5′-CUGGACCAUCGAGACAAGGUAdTdT-3′.

### Western blots

Protein samples were harvested following treatment with Laemmli buffer containing 8 M urea. Protein equivalent to 100,000 cells was loaded per lane, and subjected to SDS-PAGE before transfer to nitrocellulose (GE Healthcare).

Membranes were incubated with affinity purified, hrp-conjugated secondary antibodies against rat (Sigma), mouse (Promega) or rabbit (Promega), and visualized by enhanced chemiluminescence. The mAbs specific for Rpb1 (Pol 3.3 and 1C7) and the different CTD phosphorylations (3E10, 3E8, 4E12) were diluted in a 5% non-fat dry milk solution (0.1% Tween in PBS) at a 1:10 dilution. The rat mAbs 6D7, 1G7, 4H2 against CTD-Thr4-P were diluted in a 2% ECL Advance blocking agent solution (GE Healthcare) (0.1% Triton X-100 in PBS) at a 1:10 dilution.

### Mass spectrometry

3 × 10^8^ Raji cells were lysed in lysis buffer (50 mM Tris-HCL, pH 8.0, 150 mM NaCl, 1% NP-40 (Roche), 1× PhosStop (Roche), 1× protease cocktail (Roche)) for 30 min on ice. Samples were sonified (Sonifier 250 BRANSON, 3 × 20 cycles, output 7, duty cycle 70) and incubated on a shaker for 1 h at 4 °C. Subsequently, samples were centrifuged (10500 g, 15 min) and the supernatants were incubated with antibody-coupled sepharose G beads (mixture of CTD phosphorylation-specific antibodies) over night on a shaker. The beads were washed three times with lysis buffer and boiled with 2× laemmli buffer (95 °C, 8 min). Immunoprecipitated proteins were separated by gel electrophoresis, stained with Coomassie and bands were excised and digested as described before^59^,^60^ with minor modifications. For the mass spectrometry analysis, samples were injected in an Ultimate 3000 HPLC system (LC Packings) and desalted on-line in a C18 micro column (300 μm i.d. × 5 mm, packed with C18 PepMap™, 5 μm, 100 Å by LC Packings). The desalted sample was then separated in a 15 cm analytical column C18 micro column (75 μm ID homepacked with ReproSil-Pur C18-AQ 2.4 μm from Dr. Maisch) with a 40 min gradient from 5 to 60% acetonitrile in 0.1% formic acid. The effluent from the HPLC was directly electrosprayed into a LTQ-Orbitrap mass spectrometer (Thermo Fisher Scientific). The MS instrument was operated in data dependent mode to automatically switch between full scan MS and MS/MS acquisition. Survey full scan MS spectra (from m/z 300–2000) were acquired in the Orbitrap with resolution R = 60,000 at m/z 400 (after accumulation to a ‘target value’ of 500,000 in the linear ion trap). The six most intense peptide ions with charge states between 2 and 4 were sequentially isolated to a target value of 10,000 and fragmented in the linear ion trap by collision induced dissociation (CID). All fragmention spectra were recorded in the LTQ part of the instrument. For all measurements with the Orbitrap detector, 3 lock-mass ions from ambient air were used for internal calibration as described before^61^. Typical MS conditions were: spray voltage, 1.5 kV; no sheath and auxiliary gas flow; heated capillary temperature, 200 °C; normalized CID energy 35%; activation q = 0.25; activation time = 30 ms.

Maxquant 1.3.0.5 was used to identify proteins and quantify by iBAQ. Maxquant conditions were: Database, ipi.HUMANv3.68; MS tol, 10 ppm; MS/MS tol, 0.5 Da; Peptide FDR, 0.1; Protein FDR, 0.01 Min. peptide Length, 5; Variable modifications, Oxidation (M); Fixed modifications, Carbamidomethyl (C); Peptides for protein quantitation, razor and unique; Min. peptides, 1; Min. ratio count, 2. iBAQ values were log2 transformed, missing values substituted by a constant value, and ANOVA performed in the DANTE R package under R x64 2.15.3.

## Additional Information

**Accession codes**: The mass spectrometry proteomics data have been deposited to the ProteomeXchange Consortium via the PRIDE partner repository with the dataset identifier PXD: PXD003459.

**How to cite this article**: Hintermair, C. *et al*. Specific threonine-4 phosphorylation and function of RNA polymerase II CTD during M phase progression. *Sci. Rep.* **6**, 27401; doi: 10.1038/srep27401 (2016).

## Supplementary Material

Supplementary Information
