# Supplementary material for: Specific threonine-4 phosphorylation and function of RNA polymerase II CTD during M phase progression
Source: Sci Rep. 2016 Jun 6;6:27401. doi: 10.1038/srep27401 (PMC4893663; doi:10.1038/srep27401)
Supplement: Supplementary Information [file srep27401-s1.pdf]

Supplementary Figures

Specific threonine-4 phosphorylation and function of RNA polymerase II CTD during M phase progression

Corinna Hintermair[1§], Kirsten Voß[1], Ignasi Forné[2], Martin Heidemann[1], Andrew Flatley[3], Elisabeth Kremmer[3], Axel Imhof[2], Dirk Eick[1§]

[1]Department of Molecular Epigenetics, Helmholtz Center Munich, Center of Integrated Protein Science (CIPSM), Marchioninistrasse 25, 81377 Munich, Germany;
[2]Biomedical Center Munich, Center of Integrated Protein Science (CIPSM), ZFP, Großhaderner Strasse 9, 82152 Planegg-Martinsried, Germany;
[3]Institute of Molecular Immunology, Helmholtz Center Munich, Marchioninistrasse 25, 81377 Munich, Germany.

[§]Corresponding authors:
Corinna Hintermair, PhD

Department of Molecular Epigenetics
Helmholtz Center Munich
Center of Integrated Protein Science Munich (CIPSM)
Marchioninistr. 25, 81377 Munich, Germany.
Tel: 0049-89-3187-1529
Fax: 0049-89-3187-1200
Email: corinna.hintermair@helmholtz-muenchen.de

Dirk Eick, PhD
Department of Molecular Epigenetics
Helmholtz Center Munich
Center of Integrated Protein Science Munich (CIPSM)
Marchioninistr. 25, 81377 Munich, Germany.
Tel: 0049-89-3187-1512
Fax: 0049-89-3187-1200
Email: eick@helmholtz-muenchen.de

a **b**

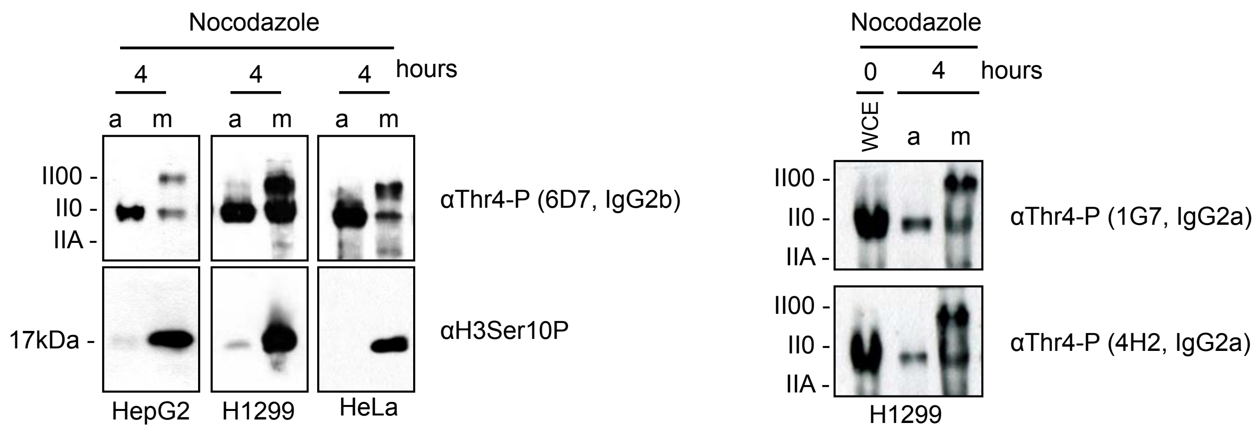

c

Phospho-peptide		Phospho-site	αThr4-P 6D7	αThr4-P 1G7	αThr4-P 4H2
CTD-1	YSPTSPSYSPTSPSC	-	-	-	-
CTD-2	YSPTSPSYSPTSPSC	T4	+++	+++	+++
CTD-3	YSPTSPSYSPTSPSC	T4,S5	-	-	-
CTD-4	YSPTSPSYSPTSPSC	S5	-	-	-
CTD-5	YSPTSPSYSPTSPSC	S7	-	-	-
CTD-6	YSPTSPSYSPTSPSC	Y1	-	-	-
CTD-7	YSPTSPSYSPTSPSC	S2	-	-	-
CTD-8	YSPTSPSYSPTSPSC	S5,S2	-	-	-
CTD-9	SPSYSPTSPSYSPTC	S2,S5	-	-	-
CTD-10	YSPTSPSYSPTSPSC	Y1,S2	-	-	-
CTD-11	YSPTSPSYSPTSPSC	S5,S7	-	-	-
CTD-12	YSPTSPSYSPTSPSC	S7,S2	-	-	-
CTD-13	YSPTSPSYSPTSPSC	Y1,T4	+++	+++	+++
CTD-14	YSPTSPSYSPTSPSC	Y1,S5	-	-	-
CTD-15	YSPTSPSYSPTSPSC	T4,Y1	+++	+++	+++
CTD-16	YSPTSPSYSPTSPSC	S5,Y1	-	-	-
CTD-17	YSPTSPSYSPTSPSC	S7,Y1	-	-	-
CTD-18	YSPTSPSYSPTSPSC	S2,T4	+	+	+
CTD-19	YSPTSPSYSPTSPSC	T4,S7	+++	+++	+++

d

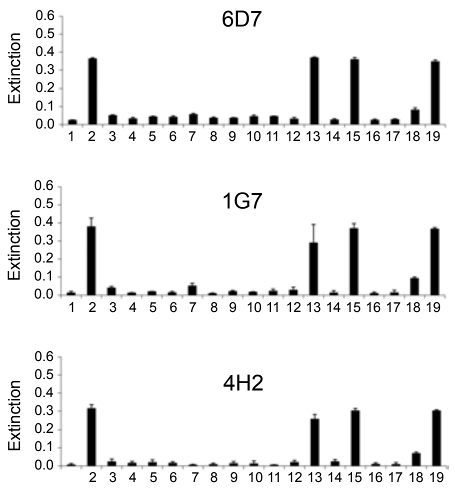

e

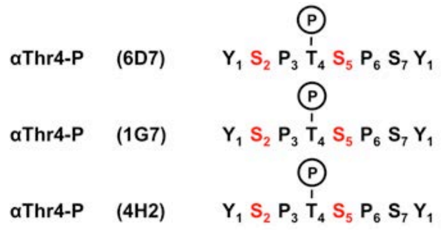

Supplementary Figure 1, related to Fig. 1 Induction of a slow migrating Pol II00 form in M phase cells. Western blot analysis of extracts of human cell lines (HepG2, H1299, HeLa) with a (**a**) Thr4-P-specific mAb (6D7) or (**b**) H1299 cell extracts with the Thr4-P-specific mAbs (1G7 and 4H2) 4 h after nocodazole (20 ng/ml) treatment. II0 and IIA designate the hyper- and hypophosphorylated forms of the large subunit Rpb1 of Pol II. II00 represents the slower migrating Thr4-P-specific Pol II form. H3Ser10-P served as a marker for mitotic cells. WCE, whole cell extract. (**c**) Survey of synthetic phospho-peptides used for characterization of CTD-specific monoclonal antibodies (mAbs). Peptide CTD-2 was used to immunize rats and identify Thr4-P-specific mAbs (6D7, 1G7 and 4H2). The binding specificity of Thr4-P-specific mAb 4H2 and of previously generated mAbs 6D7 and 1G7 was determined by an enzyme linked immunosorbent assay (ELISA) using a panel of 19 CTD peptides with different combinations of phosphorylated amino acids. Phosphorylation of amino acids adjacent to the phospho site used for immunization, inhibited (-), did not inhibit (+++), or inhibited binding of mAbs to various degrees (++, +). (**d**) Quantitative ELISA data for CTD peptides 1-19. Reactivity below 0.05 indicates background. (**e**) Overview of conditions of phospho-CTD recognition by mAbs. Red amino acids (Ser2, Ser5) indicate inhibition of mAb binding when phosphorylated. Phosphorylated amino acids coloured black (Tyr1, Ser7, Thr4) did not inhibit mAb binding. Error bars show standard deviation of three experiments.

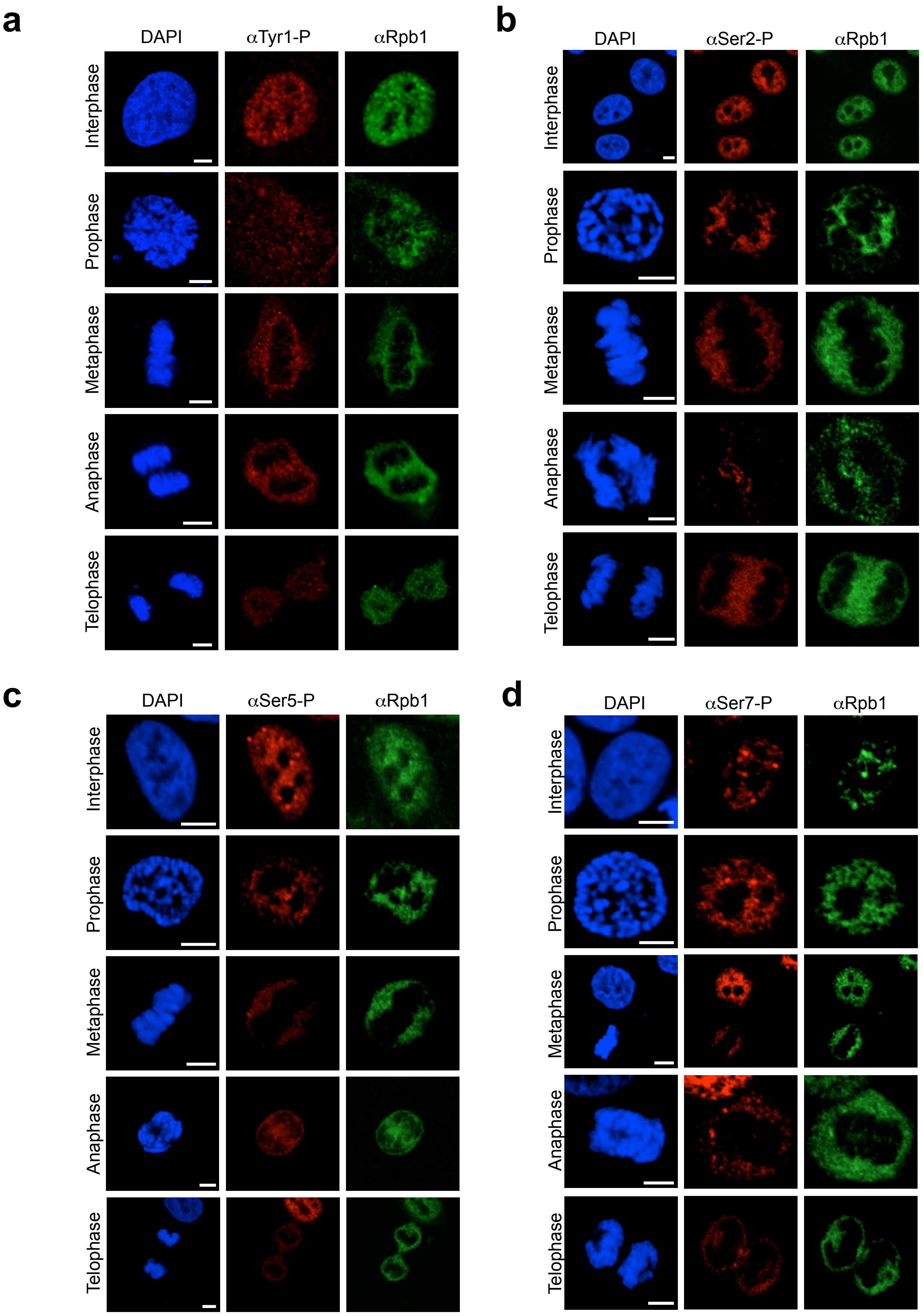
a
DAPI
αTyr1-P
αRpb1
Interphase
Prophase
Metaphase
Anaphase
Telophase
b
DAPI
αSer2-P
αRpb1
Interphase
Prophase
Metaphase
Anaphase
Telophase
c
DAPI
αSer5-P
αRpb1
Interphase
Prophase
Metaphase
Anaphase
Telophase
d
DAPI
αSer7-P
αRpb1
Interphase
Prophase
Metaphase
Anaphase
Telophase

Supplementary Figure 2, related to Fig. 2 Distribution of Pol II with specific CTD modifications during the cell cycle. Immunofluorescence images of CTD modification-specific mAbs (red) Tyr1-P (**a**), Ser2-P (**b**), Ser5-P (**c**), Ser7-P (**d**) with Rpb1 (Pol3.3; green) and DNA (4′,6-diamidino-2-phenylindole; DAPI) in HeLa cells. Representative images of cell cycle chromosomes are shown. Scale bars, 5μm.

a

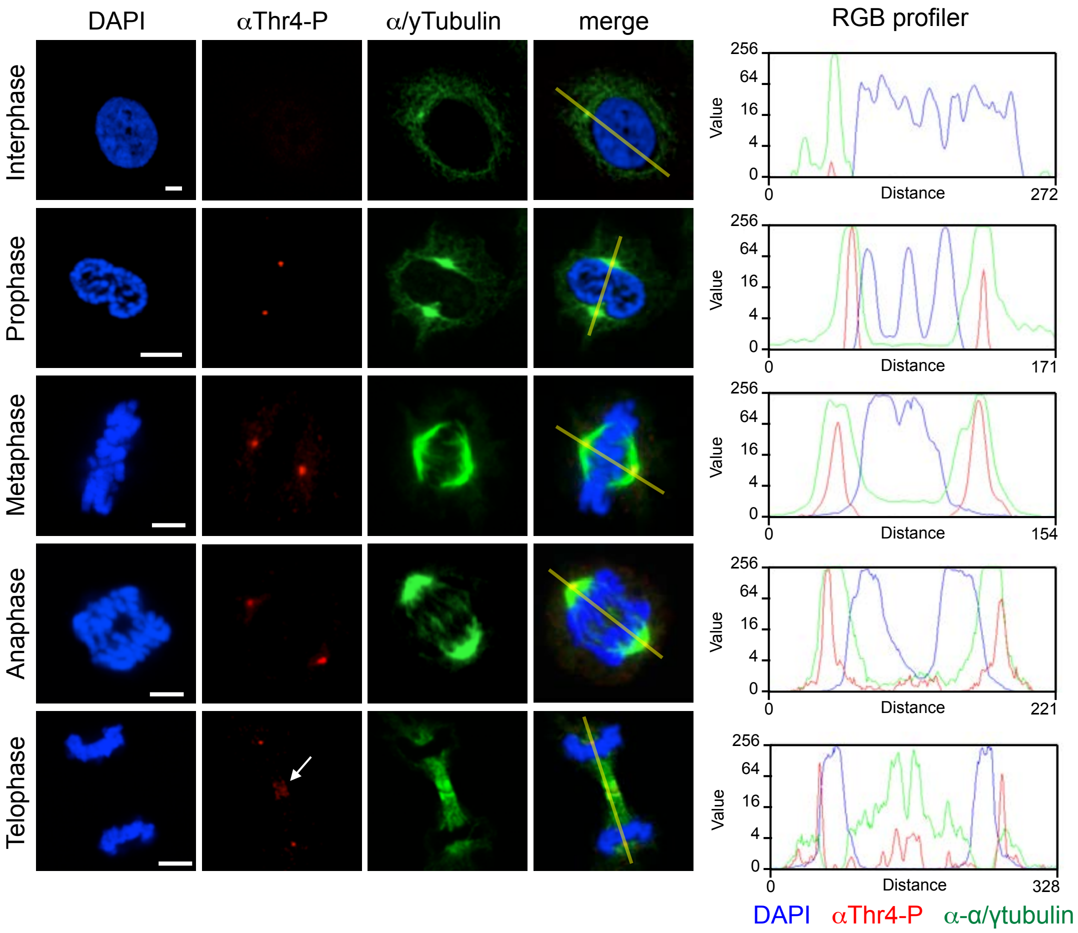

b

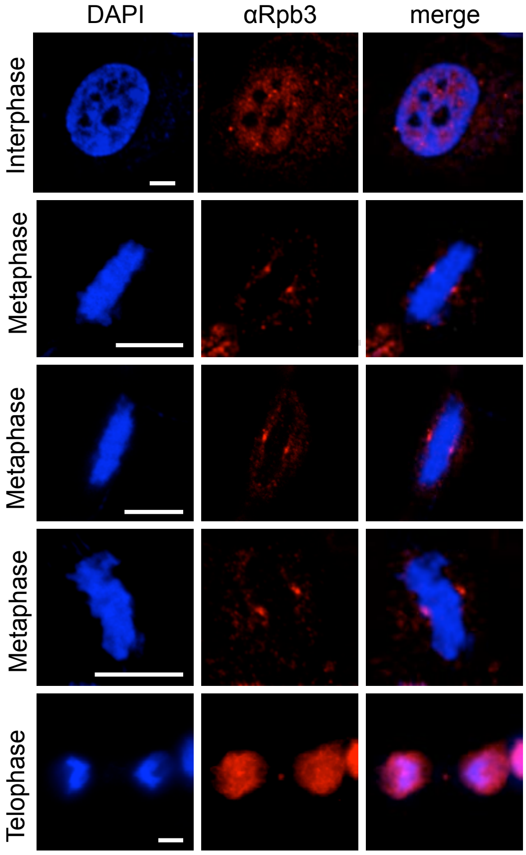

Supplementary Figure S3, related to Fig. 3 Thr4 Phosphorylated Pol II co-localizes with centrosomes in M phase. (**a**) Signals for Thr4-P (6D7, red) as well as α- and γ-tubulin (green) co-localized in each phase of mitosis. α-/γ-tubulin staining visualizes the spindle apparatus of cells (green). Representative images of each cell cycle phase are shown. Line scans visualized the localization of Thr4-P and α-/γ-tubulin signals. (**b**) Immuno-fluorescence images of Rbp3 (red) and DNA (DAPI) in HeLa cells. Representative images of cell cycle chromosomes are shown. Scale bars, 5 μm.

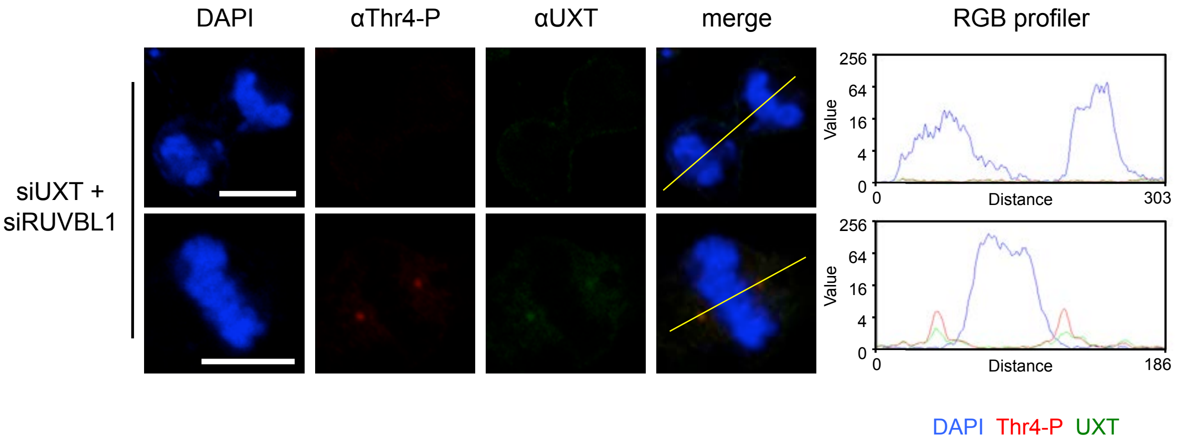

Supplementary Figure 4, related to Fig. 4 Double knockdown of RUVBL1 and UXT abolishes Thr4-P signals at centrosomes in M phase HeLa cells Immunofluorescence images of UXT (green) and the Thr4-P-specific mAb (6D7, red) in HeLa cells 48 h after siRNA double knockdown. Line scans were used to measure the relative localizations of RUVBL1, UXT and Thr4-P-specific signals. Scale bars, 5 μm.

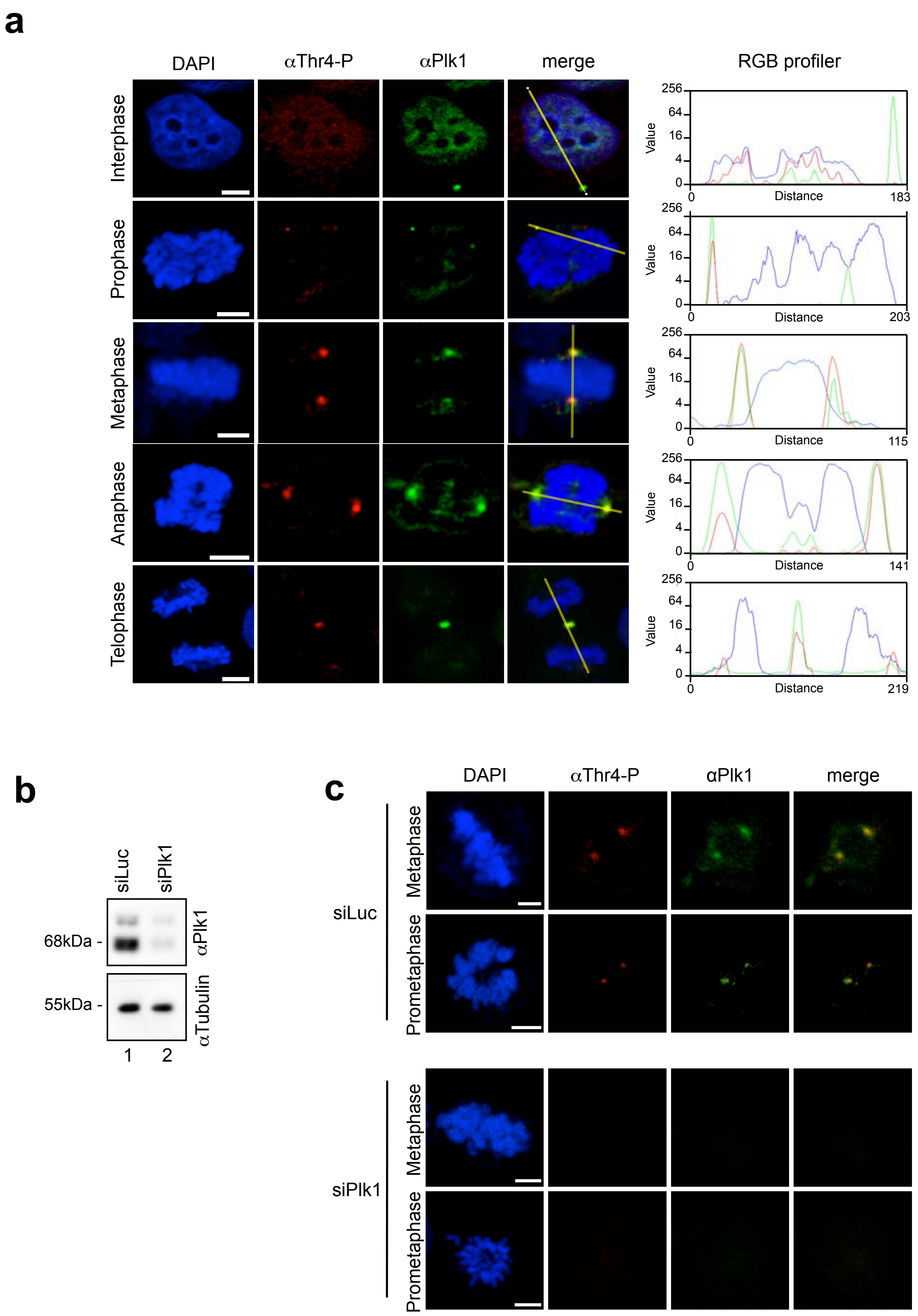
a
DAPI
αThr4-P
αPlk1
merge
RGB profiler
Interphase
Prophase
Metaphase
Anaphase
Telophase
256
64
16
4
0
Value
Distance
183
203
115
141
219
b
siLuc
siPlk1
68kDa -
55kDa -
αPlk1
αTubulin
1
2
c
DAPI
αThr4-P
αPlk1
merge
siLuc
siPlk1
Metaphase
Prometaphase

Supplementary Figure 5, related to Fig. 5 Thr4 phosphorylated Pol II co-localizes with Plk1 in M phase cells. (**a**) Co-staining of Plk1 (green) and Thr4-P (6D7; red) for each phase of the cell cycle in HeLa cells. Line scans measured the relative localization of Plk1 and the Thr4-P-specific signals. Signals from merged images were quantified using Image J 1.37V and the plug-in RGB profiler. (**b**) Western blot analysis of extracts from HeLa cells 24 h after siRNA knockdown with a Plk1-specific Ab. Tubulin served as a loading control. (**c**) Immunofluorescence images of a Thr4-P-specific mAb (6D7; red), Plk1 (green) and DNA (DAPI) in HeLa cells 24 h after siRNA transfection. Representative images of prometaphase and metaphase chromosomes are shown. Scale bars, 5μm.

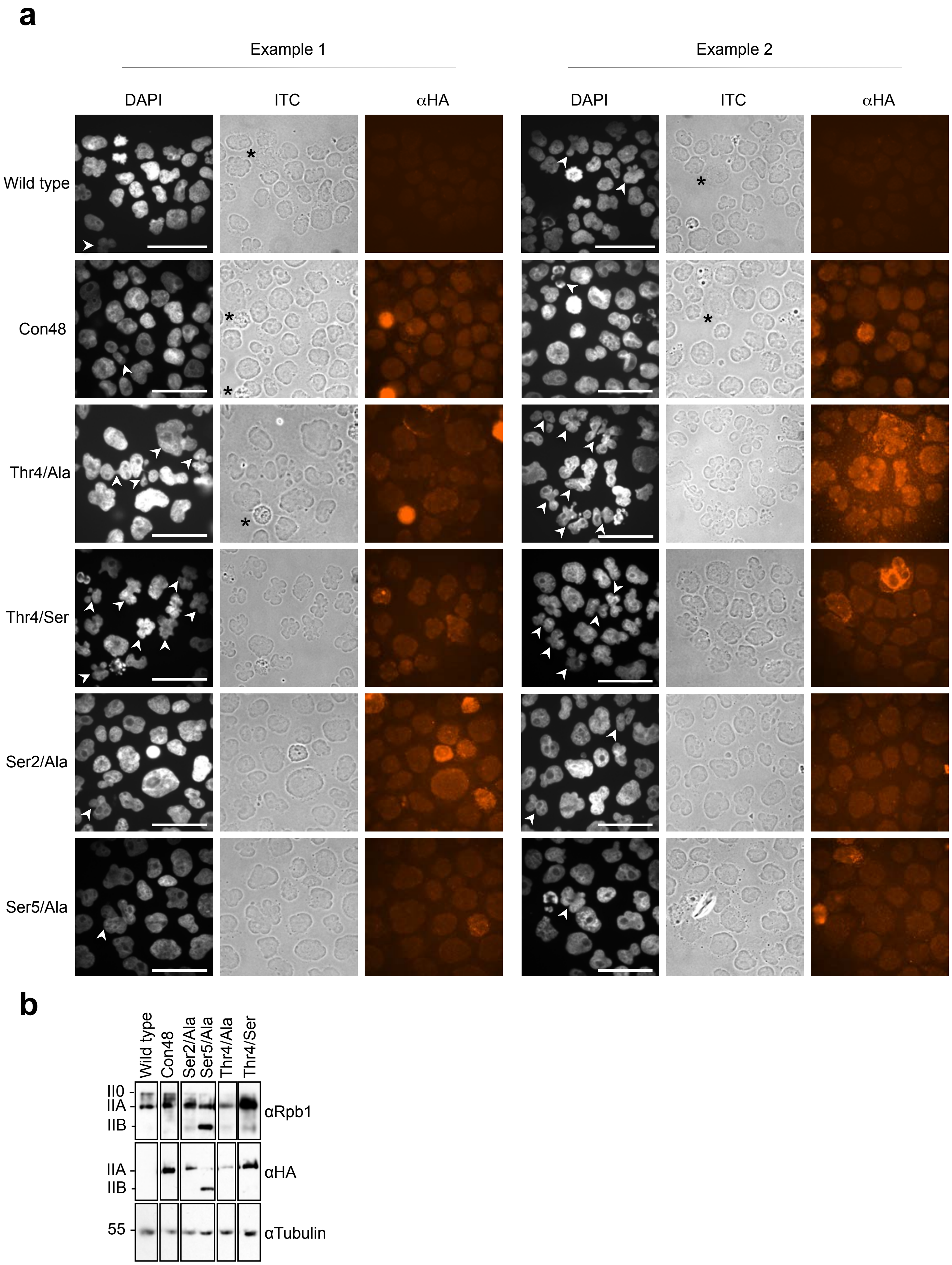
a
Example 1
Example 2
DAPI
ITC
αHA
Wild type
Con48
Thr4/Ala
Thr4/Ser
Ser2/Ala
Ser5/Ala
b
Wild type
Con48
Ser2/Ala
Ser5/Ala
Thr4/Ala
Thr4/Ser
II0
IIA
IIB
αRpb1
αHA
55
αTubulin

Supplementary Figure 6, related to Fig. 6 Impact of expression of CTD mutants on mitosis. Recombinant Rpb1 was expressed in stably transfected Raji cell lines. (**a**) 24 h after induction, the cells were plated on object slides using a cytospin and analyzed by microscopy. Immunofluorescence images of the HA mAb that recognizes the recombinant, HA-tagged Pol II and DNA (DAPI). ITC = phase contrast images. Arrowheads and asterisks indicate lobed or poly nuclei, and mitotic cells, respectively. Scale bars, 60µm. (**b**) 24h after induction, cell extracts were analyzed by western blotting with mAbs specific for Rpb1 (Pol3.3.) or HA (3F10). Tubulin served as the loading control.
